# Fast 180° magnetization switching in a strain-mediated multiferroic heterostructure driven by a voltage

**DOI:** 10.1038/srep27561

**Published:** 2016-06-08

**Authors:** Ren-Ci Peng, Jia-Mian Hu, Kasra Momeni, Jian-Jun Wang, Long-Qing Chen, Ce-Wen Nan

**Affiliations:** 1 State Key Lab of New Ceramics and Fine Processing, School of Materials Science and Engineering, Tsinghua University, Beijing, China, 100084; 2 Department of Materials Science and Engineering, The Pennsylvania State University, University Park, Pennsylvania, USA, 16802.

## Abstract

Voltage-driven 180° magnetization switching provides a low-power alternative to current-driven magnetization switching widely used in spintronic devices. Here we computationally demonstrate a promising route to achieve voltage-driven in-plane 180° magnetization switching in a strain-mediated multiferroic heterostructure (e.g., a heterostructure consisting of an amorphous, slightly elliptical Co_40_Fe_40_B_20_ nanomagnet on top of a Pb(Zr,Ti)O_3_ film as an example). This 180° switching follows a unique precessional path all in the film plane, and is enabled by manipulating magnetization dynamics with fast, local piezostrains (rise/release time <0.1 ns) on the Pb(Zr,Ti)O_3_ film surface. Our analyses predict ultralow area energy consumption per switching (~0.03 J/m^2^), approximately three orders of magnitude smaller than that dissipated by current-driven magnetization switching. A fast overall switching time of about 2.3 ns is also demonstrated. Further reduction of energy consumption and switching time can be achieved by optimizing the structure and material selection. The present design provides an additional viable route to realizing low-power and high-speed spintronics.

Multiferroic magnetoelectric heterostructures enable switching magnetization with a voltage rather than a current, dissipating much less heat^1^. Such voltage-driven magnetization switching has been achieved through the transfer of piezostrains^2^,^3^,^4^,^5^,^6^,^7^,^8^,^9^,^10^,^11^,^12^ or/and exchange coupling^13^,^14^,^15^,^16^,^17^ across the interface of the constituting magnetic and ferroelectric phases. Given that a time-invariant voltage cannot break the time-reversal symmetry of a magnetization, applying voltage alone typically induces an at most 90° magnetization switching. For example, piezostrain mediated^6^,^8^,^10^ and exchange coupling mediated^13^ voltage-driven 90° magnetization switching have both been observed experimentally in multiferroic heterostructures at zero magnetic field. Such 90° switching provides basis for the design of low-power spintronic devices such as magnetoelectric random access memories (MeRAM), which integrate a magnetic tunnel junction (MTJ) on top of a ferroelectric/piezoelectric layer^18^,^19^,^20^. A voltage-driven full 180° magnetization switching in the free layer of the MTJ would result in significantly larger electric resistance change of MTJ and hence higher signal-to-noise ratio. Pioneering experimental demonstrations include strain-mediated voltage-driven 180° switching of local magnetic domains in multilayer capacitors of Ni electrodes and BaTiO_3_-based dielectric layer^12^ and exchange-coupling-mediated voltage-driven switching of in-plane net magnetization in BiFeO_3_-based multiferroic heterostructures^16^. Theoretical proposals of achieving voltage-driven 180° switching of an almost uniform magnetization have also appeared. These are based on voltage-controlled two consecutive and deterministic 90° switching in nanomagnets with intrinsic four-fold magnetic anisotropy^21^,^22^, or precessional magnetization switching^3^,^23^,^24^,^25^,^26^,^27^,^28^,^29^,^30^.

Building on the strain-mediated voltage-driven 180° perpendicular magnetization switching suggested in ref. 3, this work computationally demonstrates an in-plane 180° magnetization switching driven by a voltage in an slightly ellipse-shaped nanomagnet on top of a 400-nm-thick (as in ref. 31) polycrystalline Pb(Zr,Ti)O_3_ (PZT) thin film as an example. The amorphous Co_40_Fe_40_B_20_ (CoFeB), which shows reasonably good magnetoelastic coupling^32^ and has been experimentally integrated with piezoelectric thin films^33^ and substrates^34^, is selected as the model magnetic material. The dimension of the amorphous CoFeB disk is 150 nm × 135 nm × 4 nm, an optimized dimension simultaneously allowing small addressing piezostrains and stable magnetization when strain is removed, see discussion later.

## Results

**Figure 1:**
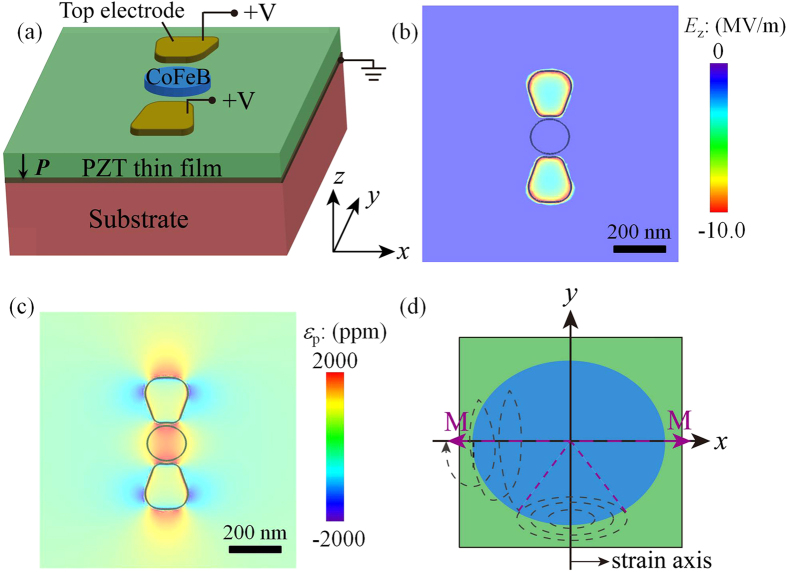
Principles of voltage-driven in-plane 180° magnetization switching. (**a**) Schematic of multiferroic heterostructure consisting of an elliptical amorphous CoFeB nanomagnet on PZT thin film. Distributions of (**b**) out-of-plane electric field (*Ez*) and (**c**) in-plane piezostrain (*ε*_p_ = *ε*_yy_ − *ε*_xx_) on the PZT surface (scale bar: 200 nm) upon applying a static 0.43-V-voltage to the two top electrodes. (**d**) Schematic illustrating the trajectory of designated in-plane 180° magnetization switching (black dashed line): an initially rightward magnetization vector **M** (purple dashed arrows) precesses around the -y-axis (or equivalently, +y-axis) under the application of *ε*_p_; Releasing the piezostrain when **M** has a negative x-component triggers a reorientation of **M** to the leftward direction by precession and damping. <*ε*_p_> represents the average of *ε*_p_ inside the central CoFeB region (marked the central ellipses in (**b,c**)).

According to our finite-element analyses (see Methods), applying identical voltages to the two top electrodes and grounding the bottom electrode (Fig. 1a) generate non-uniform out-of-plane electric-fields (Fig. 1b) and in-plane piezostrains (that is, *ε*_p_ = *ε*_yy_ − *ε*_xx_, in Fig. 1c) on the PZT film surface, consistent with existing experiments^35^. According to the simulated three-dimensional (3-D) electric field distribution (see Supplemental Figure S1), local electric fields are largely parallel to the downward polarization, indicating that polarization switching is unlikely. In addition, even the maximum local electric field (~16.1 MV/m) is well below the dielectric breakdown field of the 400-nm-PZT film (~25 MV/m from ref. 31). The average uniaxial piezostrain (<*ε*_p_>) inside the CoFeB region (the central ellipse in Fig. 1b,c) is approximately 1056 ppm. Our analysis further shows that approximately 90% of such uniaxial piezostrain is transferred to the top CoFeB magnet across the interface (see Supplemental Figure S2), because of the shear-lag induced strain relaxation^36^. However, it will be shown that the remaining uniaxial strain (about 950 ppm on average) along the *y*-axis is sufficient to overwhelm the in-plane magnetic shape anisotropy, switching the initial magnetic easy axis (EA) by 90° from the initial in-plane long axis (*x*) to the short axis (*y*).

More importantly, we propose a full 180° magnetization switching can be enabled by exploiting the dynamics of such 90° magnetic EA switching. As sketched in Fig. 1d, upon the application of the uniaxial piezostrain along the *y*-axis, the initial magnetization vector (*M*) along the +*x*-direction starts to precess around the *y*-axis (i.e., the new magnetic EA) in the film plane, and eventually will stabilize along the +*y* or −*y* direction, completing a 90° magnetization switching. If the voltage is turned off (strain will then be released) when *M* possesses −*x* in-plane component, the initial EA along the *x*-axis would reappear, and *M* would eventually stabilize along the −*x*-direction (the EA) via precession and damping.

**Figure 2:**
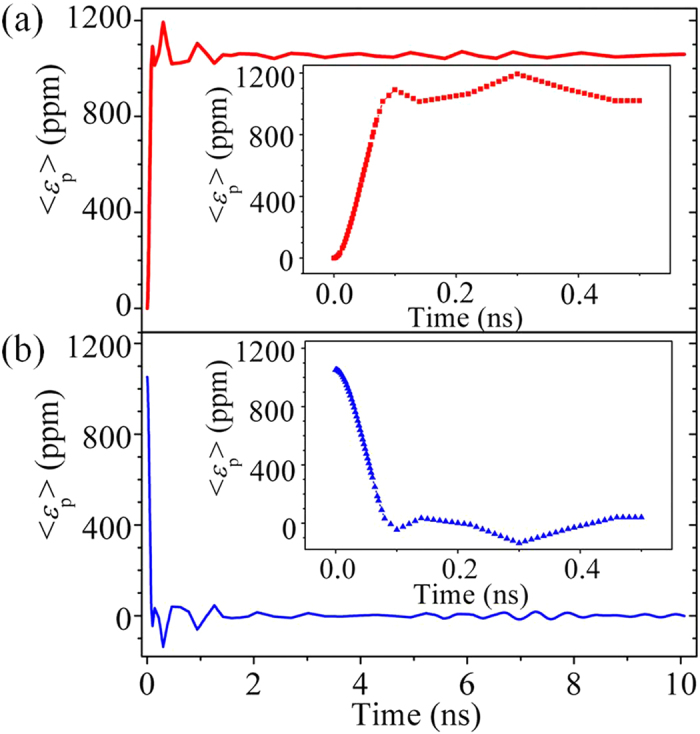
Fast, local piezostrain on the PZT film surface. The time-dependent evolution of the average piezostrain inside the CoFeB region (<*ε*_p_>) upon (**a**) applying a static voltage of 0.43 V and subsequently, (**b**), the removal of the voltage. We assume a 70-ps rise/fall time for the application/removal of the voltage (as has been experimentally achieved in ref. 37).

Thus a fast rise (release) of the piezostrain when the voltage is turned on (off) is essential to manipulate the magnetization dynamics. In this regard, finite-element models coupling linear piezoelectricity with elastodynamics (see Methods) are established. As shown in Fig. 2a, local piezostrain reaches its first peak with 0.1 ns followed by quickly attenuated oscillation around the equilibrium value of 1056 ppm. Similar feature is exhibited in the process of piezostrain release when voltage is turned off (see Fig. 2b), where the first peak also appears at about 0.1 ns. The rise and fall time of the voltage is set as 0.07 ns, the same as the experimental setup in ref. 37. We will show below that such minimum time of strain rise/release is much shorter than the time required by strain-induced magnetization switching.

**Figure 3:**
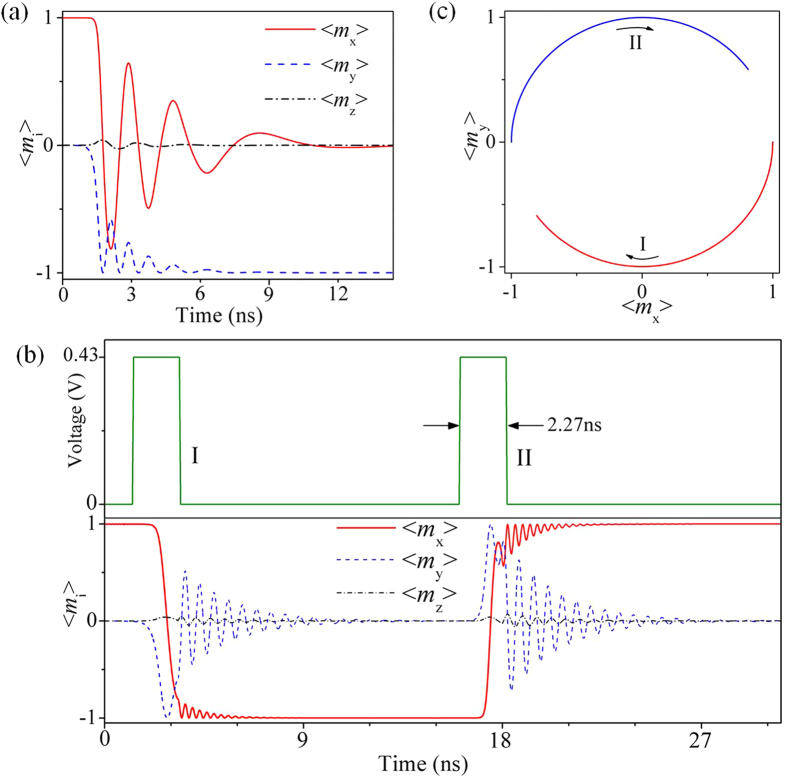
Voltage-driven fast and repeatable 180° in-plane magnetization switching. (**a**) Dynamics of the 90° in-plane magnetization switching driven by a static 0.43-V-voltage, where the <m_i_> (i = x, y, z) indicates the average magnetization of the entire nanomagnet along one Cartesian axis. (**b**) Fast and repeatable 180° in-plane magnetization switching driven by a square-wave voltage pulse with duration of 2.27 ns and an interval of 12.6 ns. (**c**) Spatial trajectories of magnetization reversal in the xy plane.

Figure 3a shows the precessional 90° in-plane switching of average magnetization from +*x*-axis (<*m*_x_> ≈ 1) to −*y* axis (<*m*_y_> ≈ −1) under the static voltage of 0.43 V, calculated from phase-field simulations (see Methods). The relatively small out-of-plane average magnetization component (that is, |<*m*_z_>|<0.1) suggests that the precession occurs largely in the film plane. If the voltage is turned off at 2.17 ns where *x*-component of the average magnetization (<*m*_x_>) reaches its negative maximum or any other stages when <*m*_x_> is negative (see Supplemental Figure S3), a 180° in-plane magnetization switching can occur. As shown in Fig. 3b, applying a square-wave voltage pulse of 2.27 ns (pulse I) can switch the magnetization by 180° from +*x*-axis (<*m*_x_> ≈ 1) to −*x*-axis (<*m*_x_> ≈ −1). The time 2.27 ns, accommodating the time required by both the strain-induced magnetization switching (2.07 ns) and the strain rise/release processes (ca. 0.1 × 2 = 0.2 ns), represents the minimum voltage pulse duration for magnetization reversal in the present design. Even shorter pulse durations can be achieved by applying larger voltage (therefore larger piezostrain), or/and by further reducing the rise/release time of piezostrain through structural optimization of both the PZT film and the top electrodes. Furthermore, applying another voltage pulse (pulse II in Fig. 3b) can switch the magnetization back to the +*x*-axis in a similar manner, where magnetization firstly precesses around the *y*-axis (see the lower panel of Fig. 3b, and more clearly, in Fig. 3c) followed by precessional relaxation. This demonstrates that the 180° in-plane magnetization switching is repeatable.

## Discussion

### Switching speed

For the present design, the pulse duration of 2.27 ns (Fig. 3) approximately represents the total time required by such piezostrain-mediated voltage-driven in-plane magnetization reversal, because both the time required by establishing/removing electric-field (~1.4 ps) and transferring the piezostrain to the top CoFeB magnet (~0.83 ps) are negligible. The former equals the time required by charging/discharging the capacitor, which is typically described by Resistance-Capacitance (*RC*) delay (that is, *t*_RC_ ≈ *R* × C). A typical resistance (*R*) of about 100 Ω (ref. 38) was used, and the capacitance (*C*) is estimated to be about 13.9 fF according to *C* ≈ *P*_r_*S*/*U*, with an area of top electrodes (*S* ~ 0.04 μm^2^), a remanent ferroelectric polarization of the 400-nm-thick PZT film (*P*_r_~15 μC/cm^2^ from ref. 31), and the magnitude of voltage pulse (*U* ~ 0.43 V). The time span for the strain transfer (*t*_p_) can be estimated according to *t*_p_ = *d*/*v* from ref. 19, where *d* is the thickness of CoFeB nanomagnet (*d* = 4nm) and *v* is the velocity of elastic wave in the solid, calculated as 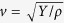 ≈ 4831 m/s using the Young’s modulus [*Y* = (*c*_11_ + 2*c*_12_)(*c*_11_ − *c*_12_)/(*c*_11_ + *c*_12_)) ≈ 187 GPa] and the mass density (*ρ* = 8 × 10^3^ Kg/m^3^) of the amorphous CoFeB.

### Energy consumption

The energy consumption per switching (*E*_con_), or the energy stored in the PZT capacitor, was estimated to be about 1.29 fJ according to *E*_con_ = 0.5*P*_r_*SU* (refs 16, 19, 39, 40, 41. The area energy consumption per switching (that is, *E*_con_/*S*) is about 0.03J/m^2^, approximately two orders of magnitude smaller than that of BiFeO_3_-film-based multiferroic heterostructure^16^.

### Energy dissipation

The energy dissipation per swtiching (*E*_dis_) was estimated to be about 0.85 fJ according to *E*_dis_ = 0.25π*C*tan(*δ*)*U*^2^ (ref. 42), where the dielectric loss tan(*δ*) should depend on the frequeny of applied voltage pulse (*f* = 1/(2.27 ns + 12.6 ns) ≈ 67.2 MHz), and the corresonding tan(*δ*) is about 0.42^43^. Note that we expect a smaller tan(*δ*) in practice, because the design employs a unipolar short-duration pulse voltage rather than bipolar ac voltage. The area energy dissipation, esimtaed as 0.021 J/m^2^ ( = *E*_dis_/*S*), is approximately three orders of magnitude smaller than that dissipated in current-driven magnetization switching. For example, an area energy dissipation of approximately 12 J/m^2^ (estimated according to (*V*^2^*t*)/(*RA*), where *V* is the voltage, *t* is the duration of current pulse, *R* is the junction resistance, and *A* is the junciton (free layer) area, from ref. 44 has been demonstrated in an Orthogonal Spin-Transfer Magneic Random Access Memory (OST-MRAM), which integrates an additional spin-polarizing layer with perpendicular magnetic anistropy to apply larger initial spin-tranfer-torque on the magnetization in the free layer^44^,^45^. The magnetizaton reversal in such OST-MRAM is also precssional^44^, and can be completed in sub-nanoseconds^45^.

### Switching energetics

**Figure 4:**
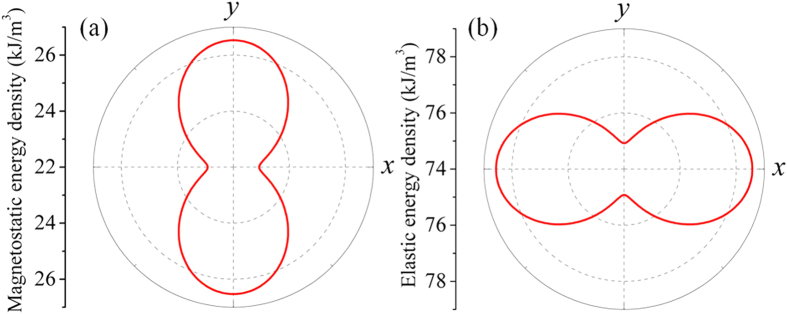
Energy analyses. Polar plots of (**a**) the magnetostatic and (**b**) the elastic energy density of the elliptical CoFeB with a dimension of 150 nm ×135 nm × 4 nm. A 1056-ppm average uniaxial piezostrain <*ε*_p_> was applied along the y-axis while the average magnetization vector was rotated by 360° in the film plane (0° represents +*x*-axis).

Figure 4a,b show the polar plots of magnetostatic and elastic energy densities of the CoFeB elliptical nanomagnet, respectively, calculated from phase-field simulations. The magnetostatic energy favors a magnetic easy axis along the *x*-axis (long axis), with a potential barrier of about 3609 J/m^3^ (Fig. 4a) while the elastic energy favors an easy axis along the *y*-axis due to positive magnetostriction, with a potential barrier of about 3644 J/m^3^. Thus the magnetostatic anisotropy can be overwhelmed by the elastic anisotropy, triggering a 90° easy axis switching from *x*-axis to *y*-axis in the CoFeB. Given the similar magnitudes of the magnetostatic and elastic potential barriers, the present <*ε*_p_> of 1056 ppm represents the critical magnitude of the piezostrain pulse (denoted as *ε*_cr_) for the easy axis switching and thereby the magnetization reversal.

### Size scaling

**Figure 5:**
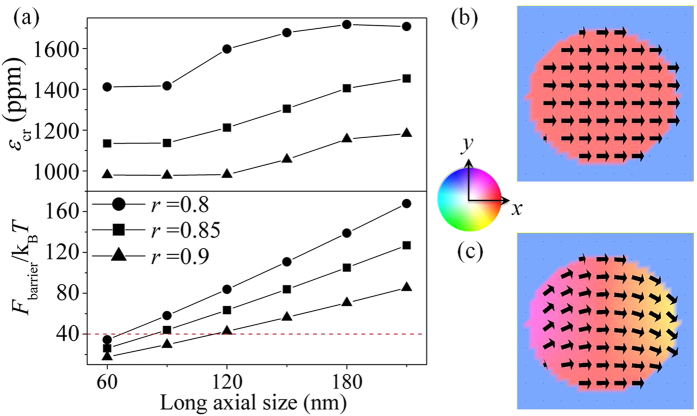
Scaling the in-plane dimension of the elliptical CoFeB. (**a**) The critical magnitude of pieostrain pulse (ε_cr_) for enabling magnetization reversal and thermal stability factor as a function of the in-plane dimension of the CoFeB nanomagnets. The aspect ratio *r* = (short-axis length)/(long-axis length). The 4-nm-thickess remains unchanged. Surface magnetization distributions (top view) of the CoFeB nanomagnets with the same aspect ratio but different in-plane dimensions: (**b**) 60 nm × 54 nm; and (**c**) 210 nm × 189 nm. The arrows and the background color (see the color wheel) both represent the orientations of local magnetization vectors.

Figure 5a shows how the scaling of in-plane dimension influences the *ε*_cr_ (see the upper panel), and in the bottom panel, the room-temperature (*T* = 300 *K*) thermal stablity factor (*F*_barrier_/*k*_B_*T*). Here the energy barrier *F*_barrier_ equals the magnetostatic potential barrier (e.g., the barrier between *x* and *y* axes shown in Fig. 4a) multiplied by the volume of the magnet, and *k*_B_ is the Boltzmann constant. A thermally stable magnetic state typically requires a minimum stability factor of 40 (ref. 19), as marked by the dashed line. For a fixed dimension of the in-plane long axis, reducing the length of the short axis decreases the aspect ratio (*r*). This leads to stronger anisotropy in magnetostatic energy density (that is, deeper potential barrier) and thereby enhanced thermal stability. The *ε*_cr_ for overcoming the energy barrier rises accordingly. On the other hand, for a fixed aspect ratio, scaling up the elliptical nanomagnet (represented by the increasing length of long-axis) also enhances the thermal stability. However, this is purely due to the increase in volume and the *ε*_cr_ remains virtually unchanged as long as the nanomagnet remains as a uniform domain (e.g. see Fig. 5b). The increased heterogeneity of in-plane magnetization appears at large dimensions, for example, the leaf-like domain shown in Fig. 5c. This will also increase the *ε*_cr_, similarly to our previous report of pulse-voltage-driven perpendicular 180° magnetization switching^3^.

## Summary

By combining finite-element analysis and phase-field modeling, we have demonstrated a nonvolatile 180° in-plane magnetization switching driven by a unipolar pulse voltage in a multiferroic heterostructure with amorphous Co_40_Fe_40_B_20_ elliptical nanomagnet on top of PZT film. We have shown that the magnetization vector, initially along the in-plane long axis, would precess across the in-plane short axis (>90° precession) when pulse voltage is on. As the voltage is turned off, the magnetization will relax to the other direction of the long axis via damped precession, completing an 180° switching. Compared to previous reports utilizing out-of-plane magnetization precession^3^,^12^,^23^,^24^,^25^,^26^, the magnetization precession trajectory is virtually within the horizontal plane mainly due to the strong out-of-plane demagnetization. Compared to the previous experimental demonstrations^26^,^27^,^28^,^29^,^30^ of voltage-controlled precessional magnetization reversal (via voltage-controlled magnetic anisotropy) in the presence of a static magnetic field, the present design does not involve the use of any magnetic fields. Furthermore, we have shown that such in-plane 180° magnetization switching is repeatable, fast (switching time ~2.3 ns) and requires ultralow energy consumption (~1.3 fJ) and ultralow energy dissipation (~0.9 fJ). Further enhancement of switching speed and reduction of energy consumption/dissipation are possible, for example, by using a thinner piezoelectric layer or/and a piezoelectric layer with larger piezoelectricity than PZT (e.g., Pb(Mg_2/3_Nb_1/3_)O_3_-PbTiO_3_, ref. 46), or using magnetic materials with stronger magnetoelastic coupling than amorphous CoFeB (e.g., Fe_81_Ga_19_, ref. 47). We believe that these promising features will make the present design a competing alternative to spin-torque-mediated current-driven magnetization switching that underpins current spintronic device technologies.

## Methods

### Finite-element analyses

We consider polycrystalline PZT film as a model piezoelectric material system. Polycrystalline PZT film has proven by experiments^35^ to be capable of generating in-plane uniaxial (biaxial anisotropic) local piezostrains through an electrode design similarly to what has been presented here (i.e., Fig. 1a). The finite-element model for the PZT piezoelectric material is implemented in the commercial software package COMSOL Multiphysics. The static electric field (***E***) and piezostrain (***ε***) are calculated by solving the corresponding electrostatic equilibrium equation (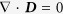) and mechanical equilibrium equation (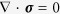) under the short-circuit boundary and mechanical clamped boundary conditions, respectively. The piezoelectric constitutive equations are,


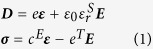


where ***D*** is electric displacement tensor, **σ** is the stress tensor, *e*^T^ is the transpose of piezoelectric stress coefficient tensor, 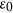 is the vacuum permittivity, 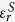 is the relative dielectric constant tensor under the mechanical clamped boundary condition, and *c*^E^ is elastic constant tensor under constant electric field. A PZT thin film of 3000 nm×3000 nm×400 nm is considered which is fixed at the bottom (by the substrate) and is free on top. To mimic a large sample, periodic boundary conditions are applied on the four surrounding side surfaces. A free tetrahedral mesh with quadratic shape functions is used for discretizing the domain. The MUltifrontal Massively Parallel Sparse direct Solver (MUMPS) is utilized to solve the governing partial differential equations. The voltage is applied to a pair of trapezoid-like top electrodes and the bottom electrode is grounded. The electromechanical properties of PZT are presented in the Supplemental Materials S5. Shape of two top electrodes is optimized to minimize the local electric field concentration and to obtain an almost uniform strain distribution in the central ellipse region.

Time-dependent simulations are performed to calculate the minimum rise (relaxation) time of generated piezostrain upon applying (removing) the voltage. A structural Rayleigh damping is considered in the PZT thin film, which is the source of energy dissipation. The governing elastodynamics equation with damping is


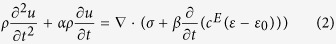


where *ρ* is density (=7.5 × 10^3^ Kg/m^3^ for PZT), *u* is the mechanical displacement, *t* is time, and *α* and *β* are the mass damping coefficient, and the stiffness damping coefficient, respectively. A PZT film typically shows a viscous damping (that is, *α* = 0 s^−1^)^48^. The stiffness damping coefficient *β* is taken as 6 × 10^−12^ s. From local piezostrain vs. time plot, the minimum rise/fall time of strain can be determined at the first strain peak after applying (removing) the voltage. Influence of stiffness damping coefficient *β* on the rise of piezostrain is discussed in the Supplemental Figure S4 for an applied voltage of 0.43 V.

The same tetrahedral mesh elements with quadratic shape functions are utilized for discretizing the space domain. The time-dependent equation (2) is solved using MUMPS direct linear solver with row preordering and pivoting in combination with the implicit time-dependent BDF solver where the time steps are chosen automatically by the solver to get best convergence. A scaled absolute tolerance of 0.001 with backward Euler initialization is utilized.

### Phase-field model

Phase-field method typically involves the use of an order parameter that changes continuously across the interface of two phases, or across a domain wall separating two domains. In the present phase-field model, local magnetization vector ***M*** = *M*_s_ (*m*_x_, *m*_y_, *m*_z_) is used as the main ‘phase-field’ order parameter, where *m*_i_ (i = *x*, *y*, *z*) represents the direction cosine (***m*** = ***M***/*M*_s_), and *M*_s_ is the saturation magnetization. Magnetic domain structures are denoted by the spatial distributions of the ***M***. The temporal evolution of the magnetization can be described by the Landau-Lifshitz-Gilbert (LLG) equation,


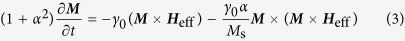


where *α* and *γ*_0_ are the Gilbert damping coefficient and the gyromagnetic ratio, respectively. Equation (3) is solved by a semi-implicit Fourier spectral method (see details in refs 49 and 50). The real time span (Δ*t*) corresponding to each normalized numerical time step (Δ*τ*) is about 0.09 ps according to 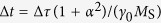, with Δ*τ* = 0.02. The effective magnetic field is expressed as 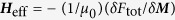, with *μ*_0_ representing the vacuum permeability and *F*_tot_ the total free energy of CoFeB nanomagnet. For the CoFeB amorphous nanomagnet grown on a ferroelectric layer, *F*_tot_ can be written as,


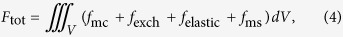


where *f*_mc_, *f*_exch_, *f*_elastic_ and *f*_ms_ are the magnetocrystalline anisotropy, exchange, elastic, and magnetostatic energy densities, respectively. The magnetocrystalline anisotropy energy is ignored due to the isotropic nature of an amorphous CoFeB nanomagnet. *f*_ms_ is relevant to the shape of the magnet and is calculated using a finite-size-magnet magnetostatic boundary condition^51^ in the present model. The magnetic exchange energy density (*f*_ex_) is calculated as 
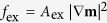
, where *A*_ex_ indicates the exchange constant. The elastic energy density (*f*_elastic_) is calculated as,


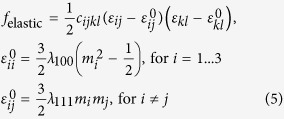


where ***ε***^0^ denotes the stress-free strain (see derivation in ref. 52) the total strain ***ε*** contains a homogeneous part ***ε***^**hom**^ and a heterogenous part ***ε***^**het**^ following Khachaturyan’s theory of microelasticity^53^. The ***ε***^**hom**^ describes the macroscopic deformation. It can arise from the lattice/thermal mismatch between the magnet and the ferroelectric layer underneath (assuming to be zero herein), or/and the applied piezostrain calculated from finite-element simulations (i.e., the average uniaxial piezostrain <*ε*_p_> in Fig. 2). The ***ε***^**het**^, showing a zero volumetric integral over the entire magnet, can be calculated according to ***ε***^**het**^ = 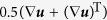, where ***u*** indicates the local mechanical displacement and is obtained by solving mechanical equilibrium equation 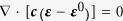.

Three-dimensional discrete grids of 32Δ*x* × 32Δ*y* × 20Δ*z* with a real grid space Δ*z* = 1 nm, and Δ*x* = Δ*y* = 5.0 nm, are used. The influence of grid space on the simulated magnetization dynamics is discussed in Supplemental Materials S6. The bottom grids of 32Δ*x* × 32Δ*y* × 11Δ*z* are utilized to describe the piezoelectric layer (PZT), the top grids of 32Δ*x* × 32Δ*y* × 5Δ*z* represent the air phase, and the remaining grids of 32Δ*x* × 32Δ*y* × 4Δ*z* in the middle are used to describe the ellipse CoFeB nanomaget with a thickness *d* ( = 4Δ*z*) of 4 nm and the air surrounding the magnet. Note that the 4-nm-thickness is smaller than the exchange length (*l*_ex_ = 
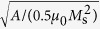
, from ref. 54) that is calculated as about 4.9 nm. Thus the local magnetization vectors should very likely be parallel to each other along the thickness direction. The shape of the ellipse nanomagnet is described by a shape function 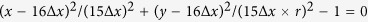 with *r* the aspect ratio. The top (air) and bottom (PZT) grids allow us to accommodate the 3-D magnetic stray field surrounding the CoFeB nanomagnet in simulations. The air phase permits creating stress-free top and lateral surfaces in the CoFeB, leading to non-uniform strain distribution in the magnet (Figure S2). Simulations for materials with different sizes can be achieved by changing the grid number or grid size (Δ*x*, Δ*y*, Δ*z*), which are used to investigate effects of the lateral size and aspect ratio on the magnetization reversal. The material parameters of CoFeB used for simulations are obtained from literatures, and the saturated magnetization, elastic constants, magnetostrictive coefficients, Gilbert damping constant, gyromagnetic ratio, and exchange constant of CoFeB nanomagnet are listed as follows: *M*_s_ = 1.0 × 10^6^ A/m (ref. 55); *c*_11_ = 2.8 × 10^11^ N/m^2^, *c*_12_ = 1.4 × 10^11^ N/m^2^, and *c*_44_ = 0.7 × 10^11^ N/m^2^ (ref. 56); *λ*_s_ = 3.1 × 10^−5^ (ref. 57); *α* = 0.005 (ref. 58); *γ*_0_ = 1.76 × 10^11^ Hz/T (ref. 59); *A*_ex_ = 1.5 × 10^−11^ J/m (ref. 60).

## Additional Information

**How to cite this article**: Peng, R.-C. *et al.* Fast 180^o^ magnetization switching in a strain-mediated multiferroic heterostructure driven by a voltage. *Sci. Rep.* **6**, 27561; doi: 10.1038/srep27561 (2016).

## Supplementary Material

Supplementary Information
