# Supplementary material for: Fast 180° magnetization switching in a strain-mediated multiferroic heterostructure driven by a voltage
Source: Sci Rep. 2016 Jun 8;6:27561. doi: 10.1038/srep27561 (PMC4897746; doi:10.1038/srep27561)
Supplement: Supplementary Information [file srep27561-s1.doc]

**Supplemental Information**

**Fast 180^o^ magnetization switching in a strain-mediated multiferroic heterostructure driven by a voltage**

Ren-Ci Peng,^1^ Jia-Mian Hu,^2*^ Kasra Momeni,^2^ Jian-Jun Wang,^2^ Long-Qing Chen,^1,2^

and Ce-Wen Nan^1*^

^1^State Key Lab of New Ceramics and Fine Processing, School of Materials Science and Engineering, Tsinghua University, Beijing, China, 100084

^2^Department of Materials Science and Engineering, The Pennsylvania State University, University Park, Pennsylvania, USA, 16802

*correspondence should be addressed to: [juh34@psu.edu](mailto:juh34@psu.edu) or cwnan@tsinghua.edu.cn

**Supplemental Figures**

**S1 Three-dimensional (3D) electric field distribution of PZT thin film using finite-element simulations**

**
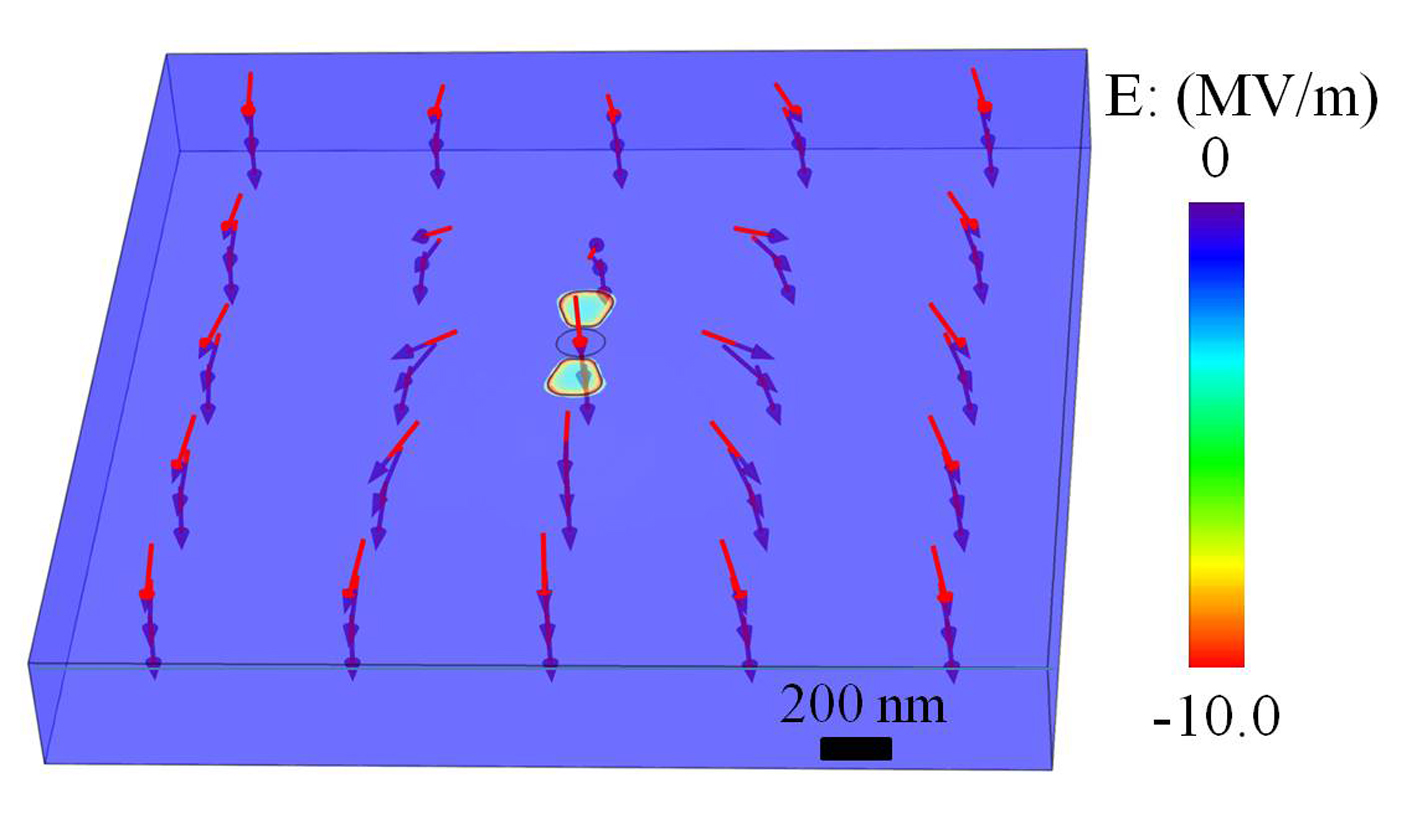
**

**Figure S1.** **Three-dimensional plot of electric field distribution in a PZT thin film (3000 nm ×3000 nm ×400 nm) under the voltage *V*_0_ = 0.43 V obtained from finite element simulations.** The color bar shows magnitude of the electric field and red arrows indicate its direction. Two trapezoid-like sections are the projection of two top electrodes (the space between two top electrodes is 150 nm and the thickness of top electrode is 5nm) and the central ellipse region is the projection of CoFeB ellipse nanomagnet with its size of 150 nm ×135 nm ×4 nm.

**S2 Strain relaxation in thin ellipse nanomagnets using phase-field simulations**

**
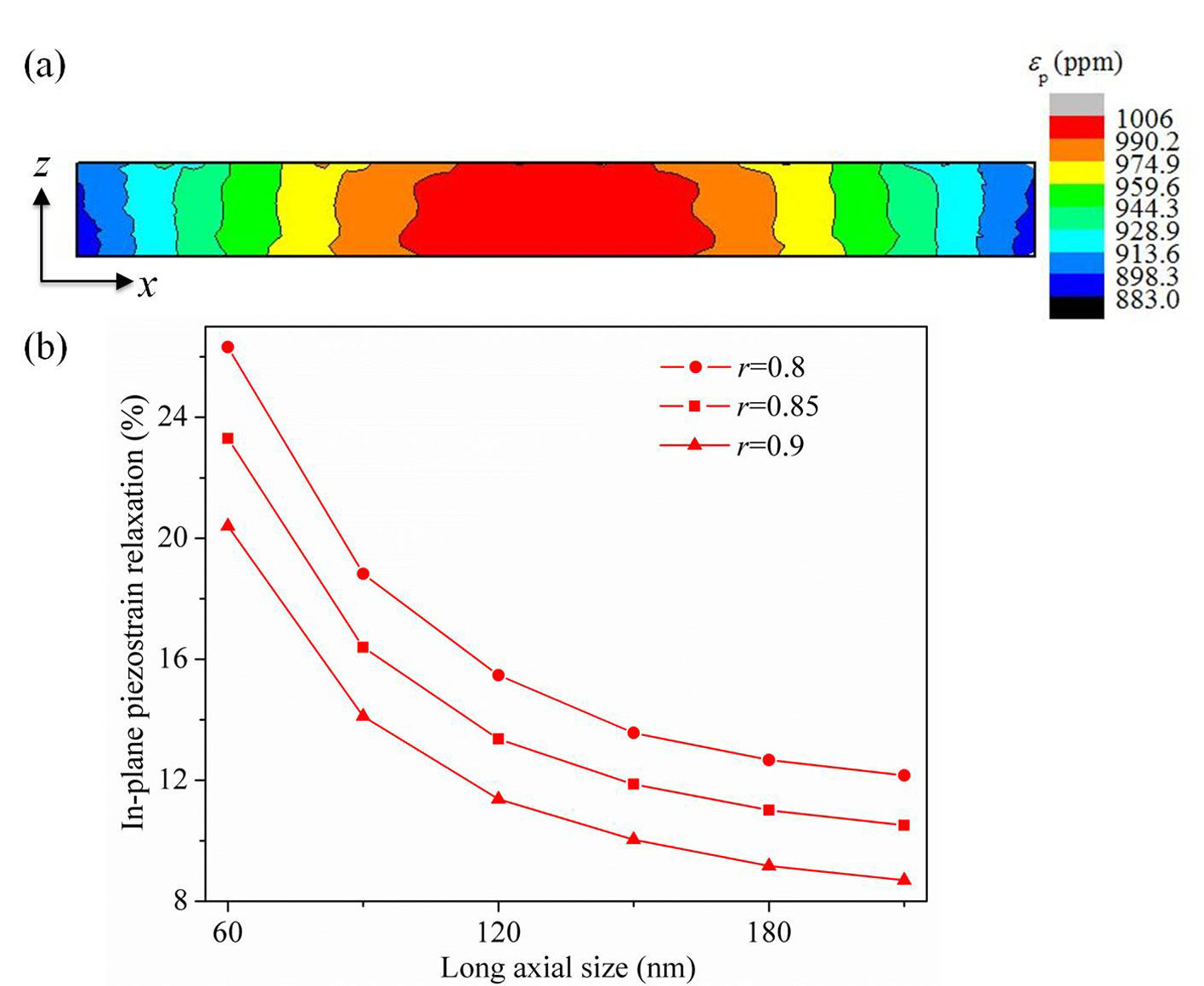
**

**Figure S2. Strain relaxation of ellipse nanomagnets.** (a) Spatial distribution of in-plane piezostrain *ε*_p_ within the *xz* plane in a CoFeB ellipse nanomagnet of 150 nm × 135 nm × 4 nm upon applying piezostrain of 1056ppm. (b) Dependence of different long axial sizes and aspect ratios (*r*=0.8, 0.85, and 0.9) on strain relaxation in ellipse nanomagnets (the 4-nm-thickess remains unchanged).

Figure S2 (a) shows the simulated in‑plane piezostrain strain (*ε*_p_) distribution within the vertical plane along the long axis across the cross section of *xz* plane, where *ε*_p_ decreases near the edge of ellipse nanomagnet. This reduction is due to boundary conditions imposed by the piezoelectric layer and its relaxation is about 10% for ellipse nanomagnet with size of 150 nm × 135 nm × 4 nm. As shown in Figure S2 (b), for the axial ratio of *r*=0.9, strain relaxation decreases significantly by increasing the lateral size (long axial sizes). Same trend is observed for other axial ratios, while the relaxation increases as the axial ratio, *r,* decreases. This decrease is due to the effect of boundary conditions. Therefore, we can reduce the relaxation by selecting a larger axial ratio, *r*, and increasing the size of long axis of ellipsoidal nanomagnet.

**S3 Phase diagram of the magnitude of voltage-induced piezostrain pulse and pulse duration**


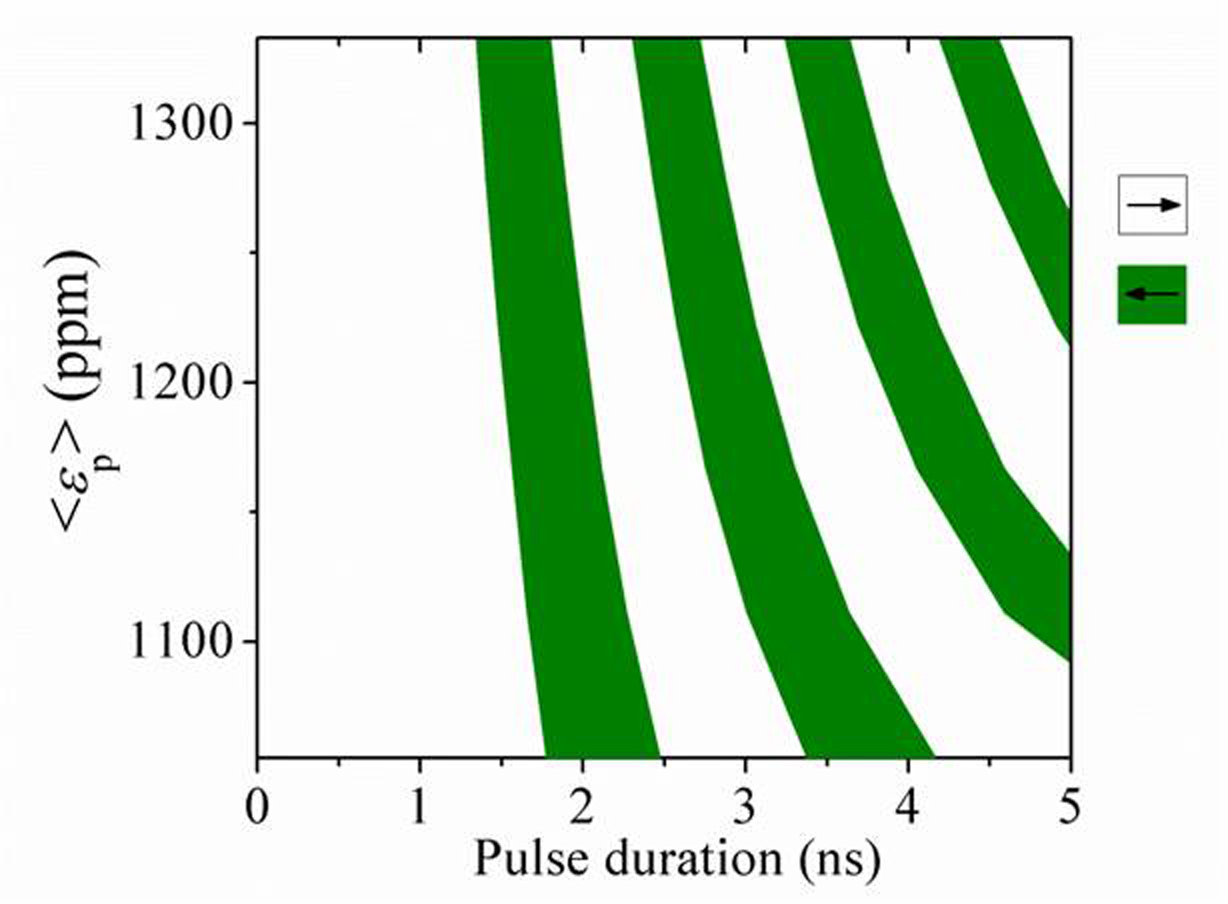


**Figure S3. Phase diagram of the magnitude of electric-field induced piezostrain pulse vs. pulse duration for in-plane uniform magnetization reversal in the amorphous CoFeB ellipse nanomagnet.** The white area denotes the magnetization vector (**m**) toward the right while the olive region is toward the left.

Duration of electrically induced piezostrain pulse is a key factor to obtain 180^o^ reversal of in-plane magnetization in the amorphous CoFeB ellipse nanomagnet. Figure S3 shows the magnitude of average in-plane piezostrain (<*ε*_p_>) versus the strain pulse duration for in-plane magnetization reversal in an ellipse nanomagnet with the size of 150 nm ×135 nm ×4 nm. When the electric-field-induced strain is less than 1056ppm, magnetic easy axis remains along the initial *x* axis rather than switches to the *y* axis and the magnetization still lies in the initial +*x* axis direction. Once larger electrostrain applied, in-plane magnetization reversal arises when the strain pulse duration reaches the desired switching regions (olive with leftward arrow), which is mainly attributed to the magnetization vector precession before reaching equilibrium (|*m*_1_| < 0.005). The oscillatory behavior of magnetization component *m*_1_ precession results in multiple possible occurrences of switching regions under the given duration of strain pulse. With piezostrain increasing, the pulse duration required for obtaining 180^o^ magnetization reversal decreases as shown in Figure S3. Moreover, the boundaries between the initial state region and the switching region are very sharp and no immediate state between them exists. This suggests that in-plane magnetization reversal can occur once magnetization component *m*_1_ is negative after removing the electric-field induced electrostrain pulse, even when the *m*_1_ becomes comparatively small and close to equilibrium after the long duration pulse.

**S4 Effect of stiffness damping coefficient *β* on the rise of piezostrain**

**
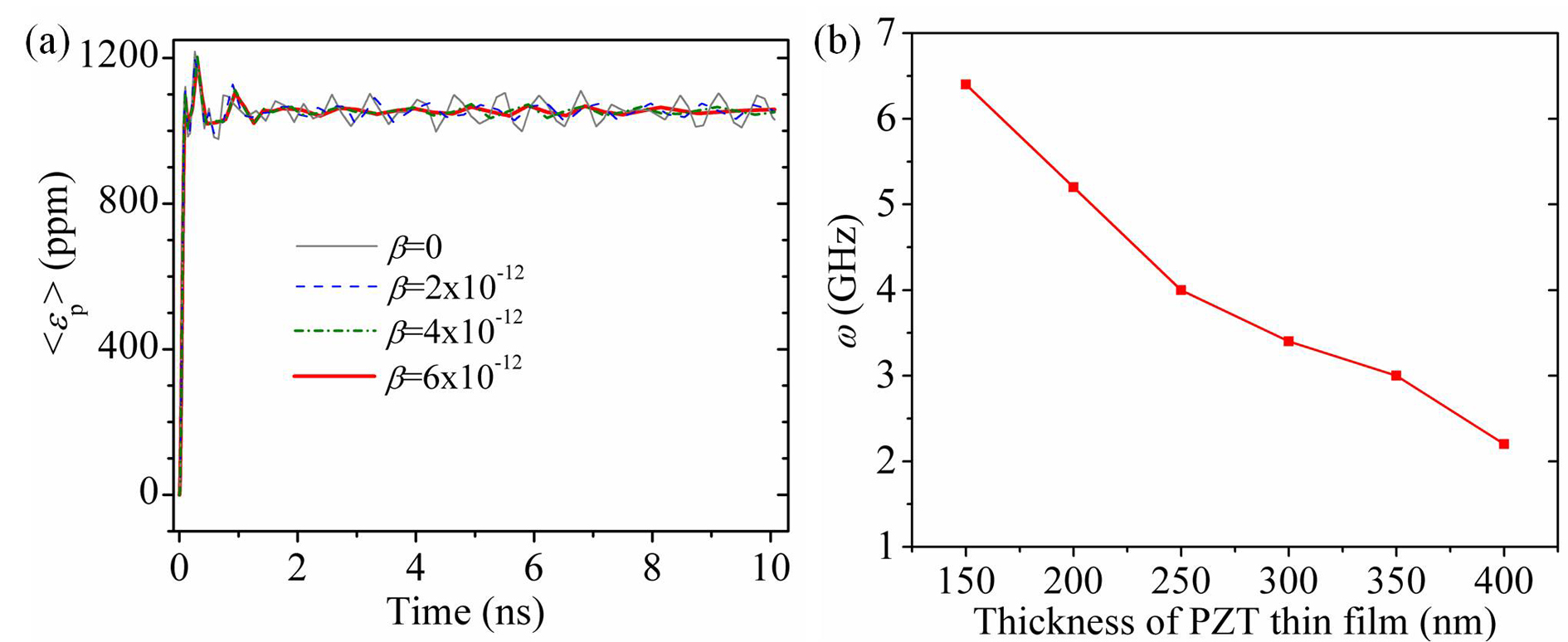
**

**Figure S4. Factor of stiffness damping coefficient.** (a) Effect of stiffness damping coefficient *β* on the rise of piezostrain under applied the voltage of 0.43 V and (b) the thickness dependence of resoance frequency (*ω*) for PZT thin film.

Figure S4a shows by increasing *β* from 0 s to 6×10^-12^ s, position of the first peak of piezostrain almost doesn’t shift and is 0.1 ns, while time to reach the final stable piezostrain value reduces by increasing *β*. Without damping (*β*=0 s), the piezostrain oscillates with a certain amplitude and will not stabilize.

Also, the stiffness damping coefficient (*β*) under an harmonic excitation can be expressed as $\beta=1/(\omega Q_{m})$[[1]](#endnote-2), where *ω* is the resonance frequency and *Q_m_* is the mechanical quality factor of PZT thin film. The influence of the piezoelectric film microstruture and film thickness on the *Q_m_* remains unknown, but it is evident that *β* depends on the resonance frequency. With thickness decreasing, the resonance frequency increases (Figure S4b) and then the corresponding *β* will decrease. Here, we assume a value of *β* (=6×10^-12^ s) for our 400-nm-thick PZT film, which much smaller than the reported value of *β* (= 3×10^-9^ s) in the 0.3-mm-thick bulk PZT ceramics[[2]](#endnote-3), given the three order of magnitude smaller thickness. Further experimental and theoretical works are requried to identiy the influence of microstructure and dimension on this stiffness damping parameter *β*, because it plays a vital role in the dynamics of coverse piezoelecric effect and hence the dynamics of piezostrain-controlled magnetization dynamics.

**S5 The tensor matrices of *c^E^*, *ε^S^*, and *e*^T^ presented based on COMSOL materials database**

The elastic constant under the short-circuit boundary condition *c*^E^ of PZT-5H is presented based on COMSOL materials database as follows:

$c^{E}=\left(\begin{matrix} 127.205,80.2122,84.6702,0,0,0 \\ 80.2122,127.205,84.6702,0,0,0 \\ 84.6702,84.6702,117.436,0,0,0 \\ 0,0,0,22.9885,0,0 \\ 0,0,0,0,22.9885,0 \\ 0,0,0,0,0,23.4742 \end{matrix}\right)\times10^{9}Pa$.

The relative dielectric constant $\varepsilon_{r}^{S}$ under the mechanical clamped boundary condition is listed as follows:

$\varepsilon_{r}^{S}=\left(\begin{matrix} 1704.4,0,0 \\ 0,1704.4,0 \\ 0,0,1433.6 \end{matrix}\right)$.

The piezoelectric stress coefficient tensor *e* is listed as follows:

$e=\left(\begin{matrix} \text{0,0,0,0,17}.0345,0 \\ 0,0,0,17.0345,0,0 \\ -6.62281,-6.62281,23.2403,0,0,0 \end{matrix}\right)C/m^{2}$.

**S6 The effect of in-plane grid space on magnetization precession**


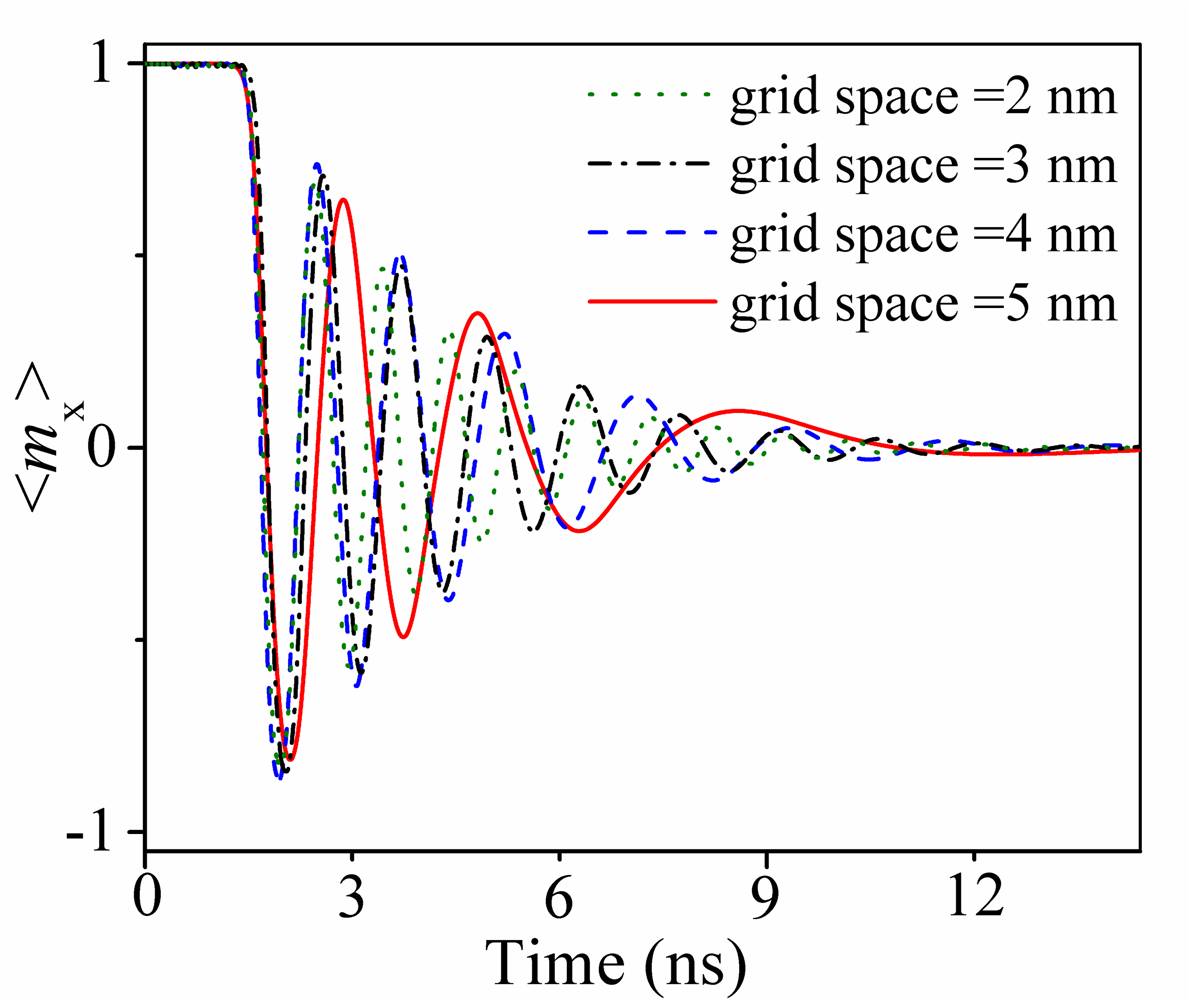


**Figure S5.** **Dynamics of the 90^o^ in-plane magnetization switching driven by a static 0.43-V-voltage in the CoFeB ellipse nanomagnet 150 nm × 135 nm × 4 nm, simulated using different in-plane grid spaces varying from 2 nm to 5 nm.**

Figure S5 shows the magnetization dynamics of a 90^o^ in-plane magnetization switching (that is, average magnetization <*m*_x_> evolves from 1 to 0) driven by a static 0.43-V-voltage in a CoFeB ellipse nanomagnet with a dimension of 150 nm × 135 nm × 4 nm. Grid spaces ranging from 2 nm to 5 nm were used. As seen, the curve calculated using the grid space of 5 nm is in general different from the others. Finer grids (such as 2 nm) typically should result in more accurate simulation results, mainly due to the more accurate description of the magnetic domain walls and the edges. However, all curves are fairly similar before <*m*_x_> reaches its first negative minimum (at around 2 ns, precisely, from 1.97ns to 2.07 ns). This is important because the voltage is turned off at the negative maximum of <*m*_x_> for realizing 180^o^ magnetization switching.

**References**

1. . Nader, G. *et al*. Effective damping value of piezoelectric transducer determined by experimental techniques and numerical analysis. *ABCM Symposium Series in Mechatronics* **1**, 271 (2004). [↑](#endnote-ref-2)
2. . Soh, C. K. *et al*. Smart *materials in structural health monitoring, control and biomechanics.* Page 67-113 (Springer, 2012).  [↑](#endnote-ref-3)
